# Dual Degrees, Dual Identities? A Longitudinal Q Methodology Study of Learner Identity Profiles in a Chinese “English + Engineering” Interdisciplinary Dual-Degree Program

**DOI:** 10.3390/bs16091656

**Published:** 2026-09-15

**Authors:** Yue Li, Meihua Chen, Honggang Liu

**Affiliations:** 1 School of Foreign Languages, Southeast University, Nanjing 211189, China; liyue2023@seu.edu.cn; 2 School of International Studies, Soochow University, Suzhou 215006, China

**Keywords:** learner identity, longitudinal design, dual-degree program, interdisciplinary education, Q methodology

## Abstract

The rapid expansion of interdisciplinary dual-degree programs in higher education poses unique challenges to students’ identity construction, particularly in highly disparate disciplinary combinations like foreign languages and STEM. Existing research on learner identity in interdisciplinary contexts largely relies on cross-sectional data, failing to capture the dynamic evolution of identities. Accordingly, this longitudinal study draws on a multidimensional framework that conceptualizes learner identity across cognitive, emotional, and behavioral dimensions and employs Q methodology and semi-structured interviews to trace the identity trajectories of 19 undergraduates in an “English + Engineering” dual-degree program across three critical time points throughout their four-year undergraduate studies. Findings reveal that at just six months after entering the program, students displayed either pluralistic exploration or an early engineering preference. After two years of learning, surging academic pressure and curriculum fragmentation were closely associated with a convergence toward an engineering-anchored identity, with some students caught in a dilemma between the two majors. Approaching graduation, the majority constructed a firm engineering-dominant identity and came to English as a competitive edge, while a highly vulnerable minority fell into a deep identity crisis. The study concludes with practical implications, calling for genuinely integrated interdisciplinary curricula and targeted psychological and academic support to mitigate identity conflicts and foster healthy interdisciplinary self-development.

## 1. Introduction

In the current era of globalization, the rapid circulation and intersection of knowledge have fundamentally reshaped higher education toward interdisciplinary restructuring, giving rise to new cultural landscapes where learners navigate overlapping disciplinary norms, value systems, and professional expectations. Such interdisciplinary integration not only aims to equip students with comprehensive professional expertise, but also offers an innovative path for disciplines to revitalize themselves in the rapidly changing context (Davies & Devlin, 2007).

Against this backdrop of a strong interdisciplinary turn with unprecedented challenges like shrinking enrollment and weaker employment prospects compared with STEM (Science, Technology, Engineering, and Mathematics) disciplines, foreign language disciplines are now inevitably impelled toward interdisciplinary restructuring (Hsu & Liang, 2021; Makhachashvili et al., 2021). In China, therefore, many schools of foreign languages in universities have rolled out “foreign language +” dual-degree programs. These programs combine foreign language with other academic disciplines, with the goals of boosting their appeal to prospective students and enhancing students’ employability (Cheng, 2022). English-related dual-degree programs are by far the most common among all these interdisciplinary pairings because English is a global lingua franca and has long occupied a mainstream position in Chinese foreign language education (Z. Chen et al., 2025; Liu, 2026; Xie, 2024).

A representative case is the combination of “English + Engineering” at S University in China. This program integrates English language training with the university’s renowned engineering strengths, aiming to cultivate interdisciplinary talents with global vision, broad-based competence and strong intercultural communication skills. The ultimate objective is to facilitate China’s participation in international technological cooperation, while also expanding students’ prospects in both the job market and postgraduate education.

Despite its potential to cultivate interdisciplinary cognition and broaden employment avenues, the dual-degree program also engenders particular identity-related concerns for student participants (Apple et al., 2020) because English and engineering are two vastly different disciplines shaped by distinct disciplinary cultures, with fundamentally different value orientations and standards for academic competence. Students in this dual-degree program must negotiate their sense of self across divergent disciplinary contexts, a process that is closely related to students’ emotions, engagement, adaptability and learning behaviors (Liu et al., 2023; Qian et al., 2026; Zhou & Liu, 2026; Zhu et al., 2026). Without effectively navigating their dual identities, students are likely to suffer from identity disorientation, which may in turn provoke anxiety, impede academic progress, and impact their psychological well-being (Y. Chen et al., 2021; Pineda, 2024; Tao et al., 2026). Therefore, exploring how these students position themselves is critical. It can not only inform targeted strategies for preventing identity crises and providing academic support for students, but also offer practical insights for the development of interdisciplinary dual-degree programs in higher education.

However, existing studies on this issue remain insufficient. Among the small body of literature examining identity construction among interdisciplinary dual-degree students, most rely on cross-sectional data collected at a single time point, which cannot adequately capture the dynamic evolution of their identities and the factors associated with such shifts. This study addresses this gap by employing Q methodology, a mixed-method uniquely suited to investigating individual subjectivity, to explore identity development among a cohort of undergraduates enrolled in the “English + Engineering” dual-degree program at S University, with data collected in the early, middle, and final stages of their undergraduate studies.

## 2. Literature Review

### 2.1. Conceptualizing Learner Identity in Dual-Degree Contexts

As a multifaceted concept, identity has been defined differently across various disciplines. Over time, theoretical perspectives to define identity have shifted from structuralist views that treat identity as a static and inherent characteristic to poststructuralist paradigms that recognize identity as a fluid and multidimensional construct shaped by historical and social contexts. From a poststructuralist standpoint, for example, identity is conceptualized not as a fixed entity, but as a highly dynamic construct. It is continuously negotiated through societal interactions and deeply molded by underlying power dynamics and prevailing ideologies (McKinney & Norton, 2024). Meanwhile, from a sociological constructivist perspective, Social Identity Theory (Tajfel & Turner, 1979) suggests that identity forms when individuals categorize themselves into social groups. They then align their cognition, attitudes, and behaviors with those groups (Stets & Burke, 2000). Researchers Ashmore et al. (2004) and Burke and Stets (2022) further proposed a model of identity with cognitive, emotional, and behavioral components. The cognitive dimension involves a person’s awareness and beliefs about their group membership. The emotional dimension relates to their sense of belonging to the group. The behavioral dimension includes actions that reflect this membership.

Drawing on this cognitive-emotional-behavioral model, the present study defines dual-degree learners’ identity as a dynamic process of recognizing, accepting, and internalizing two academic disciplines. This process operates across three interrelated dimensions: cognitively, it entails an awareness of two distinct knowledge bases and evaluative standards; emotionally, it involves a sense of affiliation with each disciplinary community; behaviorally, it requires active engagement with corresponding learning activities, and this engagement can be expressed either as balanced participation in both domains or as a clear tilt toward one.

### 2.2. Relevant Studies of Learner Identity in Dual-Degree Learning

Interdisciplinarity, merging knowledge from several disciplines to pursue an outcome that would be unattainable through just one discipline, has grown into a remarkable force in higher education (Holley, 2017). Dual-degree programs have therefore grown steadily as a common way to provide interdisciplinary training (Russell et al., 2008). In this context, learner identity, as an integral part of this interdisciplinary training (Burk & Larson, 2025; Lange, 2019), has received some attention. For example, in a study of dual-degree students in Australia, Russell et al. (2008) found that many programs simply combine subjects without real integration. These surface-level combinations, along with issues like scheduling conflicts, made it hard for students to build a secure sense of community and weaken their identity development. This concern was reinforced by Baldry et al. (2018), whose work revealed that dual-degree learning created conflicting identity positions for students. Furthermore, Pineda (2024) noted that true dual-identity formation was rare. Due to academic pressure and career expectations, most students positioned themselves in the major that offers better financial returns. On the positive side, however, some research showed positive results. Holley (2015) and Wimshurst and Manning (2017) found that some students resolved identity conflicts through self-reflection, peer teamwork, and active learning. A few even managed to maintain a balance between their two fields.

Collectively, these studies highlight the multifaceted nature of interdisciplinary identity formation among dual-degree students and identified various factors influencing this process, such as academic stress, isolated curriculum, and career aspirations. However, most of this research has relied on one-time data collection through interviews or questionnaires, overlooking the dynamic nature of identity. Identity, as defined above, is inherently dynamic and contextual. The conditions shaping a first-year student’s self-positioning, such as initial enthusiasm and low-stakes coursework, differ substantially from those facing a final-year student who must confront concrete employment decisions and postgraduate admission pressures.

Furthermore, current interdisciplinary studies predominantly focus on combinations within social sciences, law, and business. Research examining highly divergent pairings like foreign languages and engineering remains scarce. These two fields have distinct epistemological foundations, pedagogical methodologies, and disciplinary cultures. Specifically, engineering coursework typically emphasizes standardized formulas, objective data, and precise solutions to technical problems. In contrast, English studies often encourage open-ended discussions, subjective interpretations, and the exploration of diverse cultural perspectives. These daily contrasts require students to constantly switch between acting as a logic-driven problem solver and an expressive, culturally aware communicator. Navigating such different learning environments creates greater challenges for learners as they construct their identities. Longitudinal designs that track identity development among these students are therefore essential, yet rare. Accordingly, this study traces the identity development of a group of “English + Engineering” dual-degree undergraduates at a Chinese university across three key stages throughout their four-year program, guided by the following research question:

RQ: How do the students in the “English + Engineering” dual-degree program construct and negotiate their identities throughout their undergraduate studies?

## 3. Methodology

### 3.1. Q Methodology

Q methodology is a mixed-method approach uniquely suited to exploring subjectivity within small groups of participants. It integrates the strengths of qualitative and quantitative approaches by having participants (the P-set) sort a set of statements (the Q-set) according to their own subjective agreement, followed by factor analysis to identify shared configurations of subjective viewpoints based on correlations between participants’ Q-sorts. Therefore, a factor represents a shared perspective of individuals. Scholars have employed this approach to examine diverse subjective constructs such as engagement (Yao & Liu, 2026), attitude (Shaw, 2025), affective feelings (Fraschini, 2023; X. Li, 2022), and identity (Y. Li et al., 2026; Watson et al., 2025; Wu & Forbes, 2023). Q methodology typically comprises six sequential steps:

(1) Developing Q concourse. The first step in Q methodology is generally to develop a concourse, from which a subset of statements is selected to form the Q-set (Watts & Stenner, 2012). A concourse can be drawn from interviews with relevant stakeholders, as well as from the relevant literature, validated questionnaires, and other sources.

Therefore, we first conducted semi-structured interviews with a cohort of eight relevant students. These participants were recruited through the researchers’ personal contacts and referrals from student counselors. Neither the authors nor the counselors taught or assessed these students, and the counselors acted solely as neutral referral contacts. Participation was entirely voluntary and carried no implications for academic evaluation, and all interview data were anonymized. Four male and four female students were finally selected.

These students were mainly asked how they cognitively perceived, emotionally connected with, and behaviorally participated in their dual-degree programs (e.g., Do you devote more time to one major than the other?). To prevent these three dimensions from constraining the scope of statement generation, we also used open-ended prompts throughout the interviews (e.g., Are there any other feelings or perspectives you wish to share regarding your dual-degree learning?) to encourage students to freely share any learning experiences and self-perception. Interview recordings were then transcribed verbatim to extract as many statements related to the research topic as possible. Established scales and prior scholarship on identity and dual-degree learning further informed the statement pool (Russell et al., 2008; Wu & Forbes, 2023), producing a preliminary concourse comprising 47 statements.

(2) Determining the Q set. A Q-set typically comprises 40–80 statements (Watts & Stenner, 2012). A pilot Q-sorting with four students assessed the clarity, comprehensibility, and wording of the 47 items, after which two specialists in Q methodology and identity research reviewed the statement pool. Seven redundant or unclear items were removed. For instance, the statement “I do not believe I can succeed in both my majors” was removed because its negated wording was confusing to sort during the pilot stage, and its content was deemed less directly relevant to the core identity construct compared with other statements that more specifically addressed disciplinary competence and self-positioning. A final 40-statement Q set (see Appendix A) was thus established. Each statement was printed on a 90 mm × 60 mm card for the three subsequent rounds of Q sorting.

(3) Selecting participants (the P set). The participant sample was drawn from a public university situated in eastern China. The “English + Engineering” program at this university serves as a typical response to the Chinese higher education reform initiatives, which strongly advocate for the interdisciplinary integration of the humanities and STEM fields. The program requires students to simultaneously study for the English major and an engineering-related major. Upon successful completion of all credit requirements for both disciplines within a four-year period, participants are awarded a dual Bachelor of Arts and Bachelor of Engineering degree. The program began enrolling students in September 2022.

Accordingly, the potential participants were drawn from the university’s inaugural cohort of the “English + Engineering” dual-degree program (those who enrolled in September 2022). This inaugural cohort consisted of 24 students. Prior to the first Q-sort, invitations were sent to all students, and 22 voluntarily agreed to participate in this longitudinal study. However, participant attrition occurred during the tracking process: two students withdrew from the study at the second Q-sort, and one additional student withdrew from the study at the third Q-sort. While partial Q-sort data were collected from these three individuals before their withdrawal, they lacked the complete three-stage data required to map a full longitudinal identity trajectory. As a result, they were excluded from the final analysis. Consequently, the final longitudinal sample (the P set) comprised 19 students (8 females, 11 males) who completed all three stages of the study.

**Figure 1:**
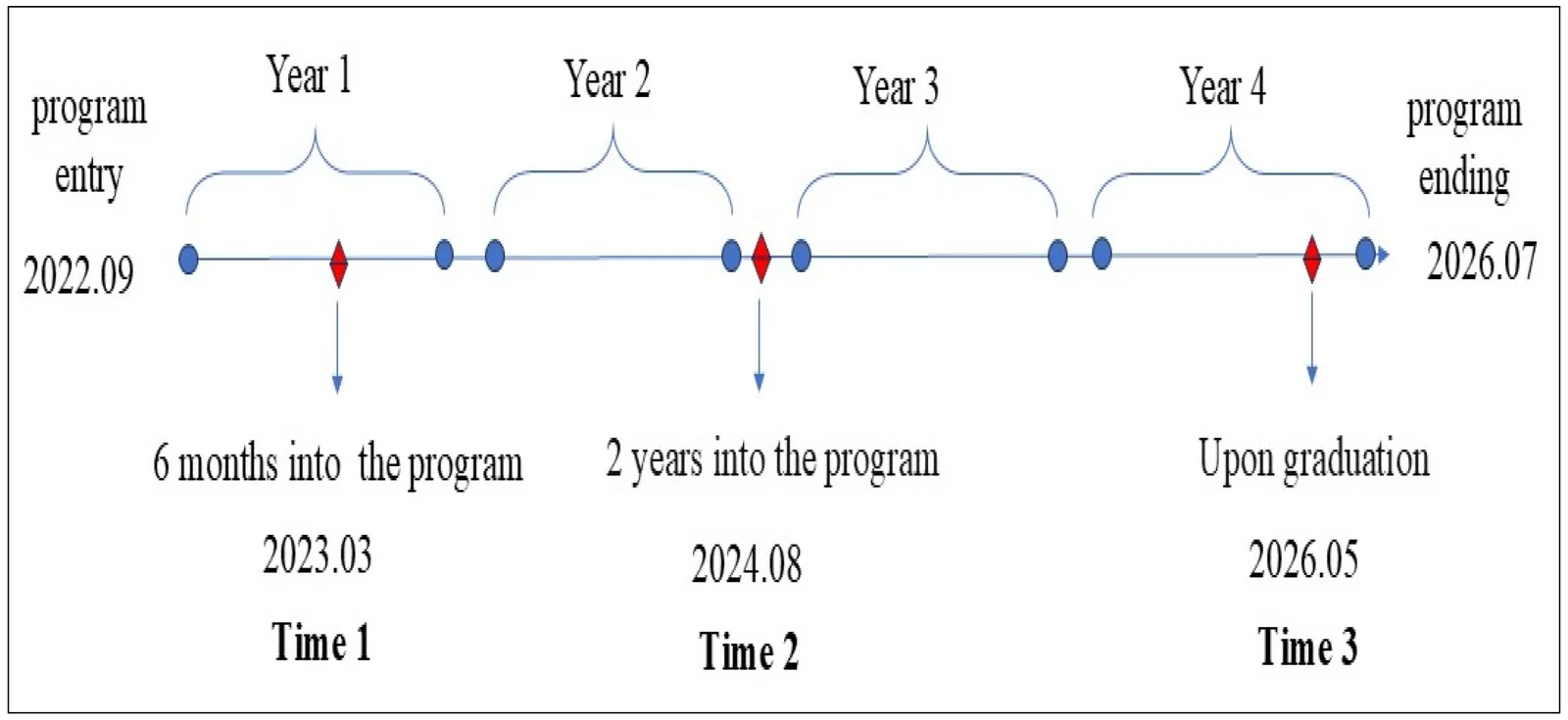
Timeline of longitudinal data collection.

**Figure 2:**
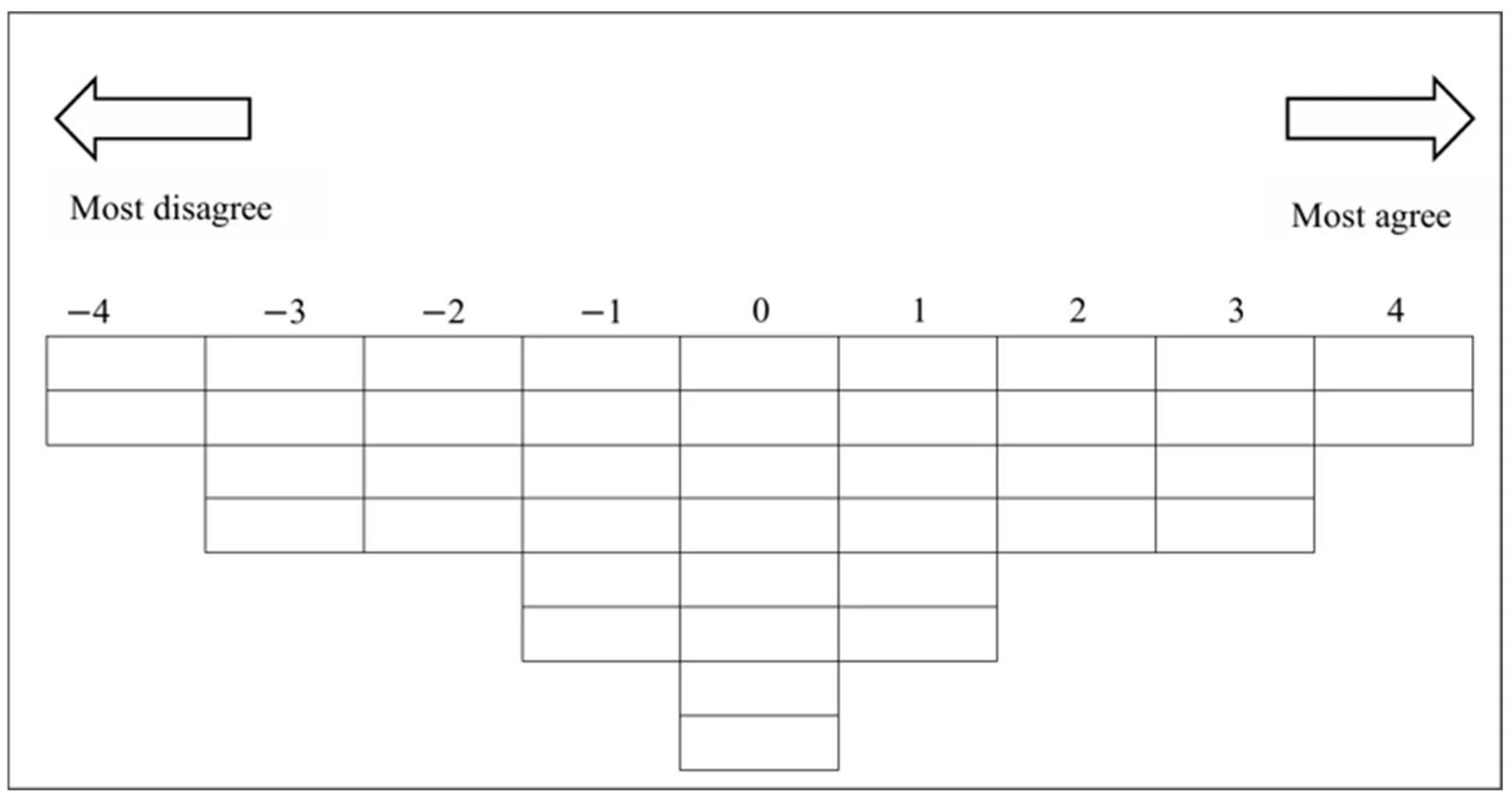
The Q sort grid.

(4) Organizing Q sorting. The participants were invited to a classroom to complete the same Q-sorting task at three time points: March 2023, August 2024, and May 2026 (please see Figure 1). At the beginning of each sorting, we provided participants with similar instructions, such as: How would you describe your position within this dual-degree program? An English major, an Engineering major, both, or neither? These prompts were intended to help participants quickly immerse themselves in the sorting task. Subsequently, participants placed the 40 cards onto a standard Q-sort grid (Figure 2) with a 9-point scale from “most disagree” (−4) to “most agree” (+4), according to how much they agreed with each statement (Watts & Stenner, 2012).

(5) Analyzing Q sorting data. Independent analyses of the three datasets were conducted utilizing the Ken-Q Analysis Desktop Edition (KADE) application. Commencing with the Time 1 dataset, a Principal Component Analysis (PCA) was executed to extract factors under the following criteria (Watts & Stenner, 2012, pp. 105–110): (a) the eigenvalue exceeds 1.0; (b) a minimum of two Q sorts with statistically significant loadings on the same factor (*p* < 0.01). The threshold for a significant factor loading at *p* < 0.01 was computed as 2.58 × (1/√*N*). *N* means the number of Q statements. Given the 40 statements used here, the corresponding threshold was 0.41; (c) the cross-product of the two highest factor loadings (regardless of the sign) exceeds twice the standard error (1/√*N*). Based on these criteria, a two-factor solution was identified. Subsequently, a varimax rotation was used to clarify each factor with statements ranking, and a two-factor structure was confirmed for Time 1. Data from Times 2 and 3 were analyzed using the same procedure. Table 1 summarizes all factors extracted across three timepoints, and detailed Q statement rankings are provided in Appendix A.

(6) Interpreting Factors. Factor interpretation followed the “crib sheet” procedure (Watts & Stenner, 2012, p. 150). We first looked at the extreme statements for each factor to get a sense of its main features. We also examined the statements that distinguished factors or were shared across them, which helped us compare the views of different times more thoroughly and dynamically. Throughout this process, the later interviews gave us additional background information that we used to name and interpret the factors. It should be noted that participants showing confounding or non-significant factor loadings (2 at Time 1, 1 at Time 2, 1 at Time 3) were not considered when interpreting factors, following standard Q methodology procedures to preserve factor purity and clarity.

### 3.2. Follow-Up Semi-Structured Interviews

To further interpret the rationales behind Q-sorting activities, this study adopted semi-structured interviews as a supplementary research method. Group interviews were conducted with each dormitory group (usually consisting of four students per dormitory) within 2–3 days of completion of Q-sorting. Students who were not in a dormitory group (i.e., those living alone) were interviewed individually.

Each interview lasted normally 60–90 min and was conducted in Mandarin. After participants had reviewed their own Q-sort records without knowing their assigned factor labels, the interview explored their most and least agreed-upon statements, distinguishing and consensus statements, and their learning and identity-related experiences. The interview prompts used are listed in Appendix B.

After obtaining informed consent from participants, we audio-recorded all interviews and transcribed them verbatim. The authors independently reviewed these transcripts to extract representative interview excerpts corresponding to each Q factor. Specifically, we focused on the transcripts of participants with relatively high factor loadings for each factor (typically those ranked within the top three). Excerpts were then selected from these representative transcripts following two clear criteria: relevance and narrative depth. For relevance, we retained excerpts that directly elaborated on the factor’s defining extreme statements (like items rated ±3 or ±4) or distinguishing statements that separated the factor from others. For narrative depth, we prioritized coherent, detailed quotes that fully captured the collective perspective shared by members of the factor group. Member-checking was then carried out, whereby the selected interview excerpts were shared with the relevant participants for their verification and feedback.

To ensure reliable English translations of the selected Mandarin excerpts, authors with English degrees and expertise in foreign language education independently translated them. The translations were then compared with the originals, and an independent bilingual expert was consulted to resolve any discrepancies and confirm semantic and contextual accuracy.

### 3.3. Research Positionality

In this study, the authors adopted a mix of insider and outsider positionality. The first author studied at the same department as participants and maintained regular contact with them throughout the tracking, which allowed full familiarity with their daily learning environment and quick rapport building. The second author was a co-initiator of this dual-degree program and knew its curriculum design and training objectives from the inside. This closeness helped us understand what participants were going through, but it also carried a risk: students might hold back from criticizing the program or expressing negative feelings. To encourage honest disclosure as much as possible, we told participants during data collection that their critical comments were valuable for improving the program and would have no bearing on any academic assessment. To keep the interpretation objective, the third author, an external researcher from another university, took on an independent critical auditing role. We cross-checked the insider readings of interview data against the third author’s independent review and against the results of the Q-sorts to guard against interpretive bias.

### 3.4. Ethical Considerations

This study adheres to stringent ethical standards to ensure participant rights. Written informed consent was obtained from all participants before data collection, with explicit explanations of the research purpose, data collection procedures, anonymous data processing rules, and the right to withdraw at any time without penalty. All personal information of participants was fully anonymized in the manuscript to protect privacy, and no identifiable personal information or portraits were presented in the text.

## 4. Findings

**Figure 3:**
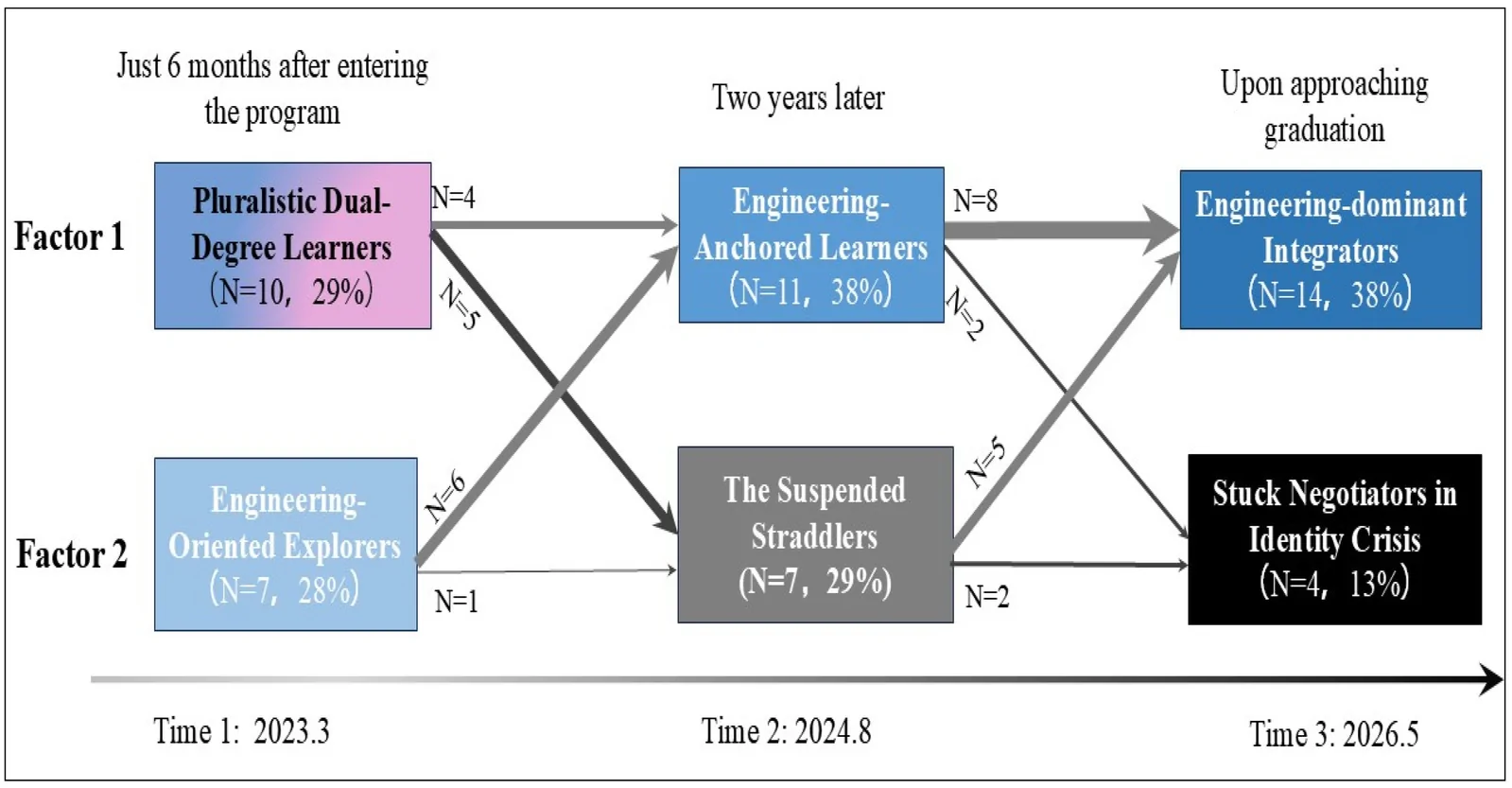
The development trajectories of identity profiles of 19 participants.

This section shows the evolving identity profiles of the 19 participants across three critical time points throughout their four-year undergraduate journey: just six months after entering the program, two years later, and upon approaching graduation. Figure 3 illustrates the identity development trajectories of the 19 research participants. Each rectangular box represents one factor, and each factor captures a shared learner identity perspective. Every box is labeled with the number of participants defining this factor and explained variance. Arrows indicate identity transition pathways between adjacent stages, and the numbers on the arrows denote the number of participants who experienced identity shifts. Detailed Q-factor assignments for all participants at each time point are provided in Appendix C.

The subsequent text outlines each factor through a descriptive narrative, which is further corroborated by quotes from the participant interviews.

### 4.1. Time 1: Just Six Months After Entering the Program

During the initial stage in the middle of their first academic year, two factors emerged from the Q-sort analysis. These factors represented two pathways through which freshmen conceptualized their identities upon entering the program.

#### 4.1.1. Factor 1: Pluralistic Dual-Degree Learners

Factor 1 accounted for 29% of the total variance, with 10 participants loading significantly onto this perspective. This viewpoint strongly embraced the interdisciplinary model (Q Statement 1, Q Ranking +4, henceforth, only statement numbers are shown), expressing high confidence that combining English and engineering satisfied their multifaceted academic interests (3, +4), enhanced their complex problem-solving capabilities (31, +3), and strengthened their core competitiveness in the job market (19, +3). Consequently, these learners clearly defined themselves as dual-degree individuals (6, +3) and rejected single-major labels (4, −4; 5, −3).

Interview data revealed participants’ pre-enrolment considerations when selecting the dual-degree program. One participant reflected:

“From the very start, choosing this dual-degree program felt like taking a risk. We were the first cohort, after all, with no available data on employment or graduate study to refer to. Even so, in such a rapidly evolving world, learning extra skills can never be a bad thing. The combination of arts and science studies also shapes my personality in diverse ways and broadens my range of interests, helping me develop a more open, adaptable personality.”

(Q4)

Another participant emphasized the competitive advantage of possessing dual expertise in modern professional environments.

“Many people argue that pairing English with a technical specialization gives you a competitive edge, while proficiency in English alone is no longer sufficient. I fully agree, as employers are clearly prioritizing interdisciplinary talents in today’s job market.”

(Q18)

These interview excerpts illustrate that their dual-degree identities were shaped jointly by internal passions and external pragmatic considerations. Motivated by this dual set of motivations, they actively sought to break down disciplinary divides and develop a unified and cohesive professional self.

#### 4.1.2. Factor 2: Engineering-Oriented Explorers

This factor accounted for 28% of the variance, defined by 7 participants. Although participants sharing this view acknowledged the general value of interdisciplinary study (1, +2; 31, +2), they placed greater emphasis on their engineering identity (5, +4) and attached less importance to their identity as English majors (4, −4). Accordingly, they directed their attention and energy predominantly toward engineering classes and industry information (16, +3; 11, +4; 13, +3). English was framed not as an equal academic discipline, but as a secondary utility tool serving engineering goals. One participant explained this pragmatic stance during the interview:

“To me, English is just a tool rather than a standalone career skill. Basic communication and the ability to read technical papers are sufficient, and my core competitive strength still lies in my engineering expertise.”

(Q10)

Beyond such pragmatic considerations of the two disciplines’ utility, a participant also expressed an innate, long-standing personal preference for engineering:

“I’ve actually always leaned more toward science and engineering subjects in my high school. To skip the Gaokao (China’s national college entrance examination, which is usually highly competitive), I gained university admission through the English recommended admission track (a pathway that spares outstanding English students from taking Gaokao). I originally planned that if I could only enroll as an English major, I would transfer to a science and engineering program at the start of my sophomore year.”

(Q19)

Holding this mindset, they struggled to regard the two disciplines on an equal footing (24, +3). Their learning resources and energy consistently leaned toward engineering. They invested far less effort in English coursework (10, −3), a pattern that gradually solidified their hierarchical identity framing, with engineering serving as the primary discipline and English acting only as secondary support.

### 4.2. Two Years Later

By the two-year midpoint of their undergraduate studies, clear shifts emerged in participants’ identity profile. They showed a stronger convergence toward the engineering major, alongside growing uncertainty between two majors.

#### 4.2.1. Factor 1: Engineering-Anchored Learners

This factor explained 38% of the variance with 11 participants. After two years of learning, the preference for engineering developed into a dominant identity (5, +4) within this perspective. Participants sharing this viewpoint expressed strong alignment with their engineering major and devoted nearly all their after-class study time to engineering subjects (11, +4). Looking ahead, this perspective reflected an intention to pursue postgraduate studies or careers within engineering fields (34, +3; 38, +3). Meanwhile, they held growing uncertainties about how employers would perceive their dual-degree background (25, +3). One participant articulated these pragmatic worries as follows:

“Others always say having a dual degree sounds cool, but I am actually quite worried about its recognition. Since this model is relatively new, I do not know whether employers will truly value it or if they will think we just dabbled in both fields without mastering either.”

(Q9)

Additionally, another participant reported that compared with their freshman year, when they followed their inner passion for a double major, their identity formation became increasingly influenced by external factors, notably career prospects and labor market demands.

“Honestly, when I first joined the dual-degree program, my choices and how I saw myself were all driven by personal interest. I was just genuinely curious about both fields. But as coursework grew heavier each year, I gradually lost that interest-focused way of thinking. These days, how I define myself and where I direct my efforts are increasingly governed by practical career prospects and the demands of the job market, rather than my initial inner motivations.”

(Q1)

#### 4.2.2. Factor 2: The Suspended Straddlers

This factor accounted for 29% of the variance and was defined by 7 participants, representing a shared experience of struggling with disciplinary misalignment. While these students maintained a strong belief in the merits of dual-degree education (1, +3), they were deeply entangled in a sense of being pulled between two majors, uncertainties about their future (40, +4), concerns about the market acceptance of their dual degrees (25, +3), and the anxiety that ensued from these tensions (23, +3). Although they recognized the importance of English within their dual-degree portfolio (29, +4) and made practical efforts to combine it with their engineering coursework (33, +3), their actual investment of time and effort in English studies remained severely constrained (10, −4). Some students described the disconnect between expectations and reality:

“Before entering the program, I assumed that there would be some interdisciplinary integrated courses. However, the two majors were delivered entirely separately, with no substantive connection between the coursework. This turned out to be quite different from my initial expectations of what a dual-degree education would entail.”

(Q11)

“Sometimes I really feel torn, and even a bit anxious. I know that if I do well with the “English + Engineering” combination, it will definitely give me an advantage, but I can’t help gravitating toward the more difficult engineering courses while neglecting the foreign language. This is not intentional, but gradually I have lost balance between the two disciplines and moved further away from what I initially envisioned for myself.”

(Q2)

Thus, these students appear to be suspended in a state where their cognitive beliefs diverge from their actual practices. They affirm the ideals of interdisciplinary education but struggle to find practical synergies between their two majors. They see the worth of English, yet are pressured into neglecting it, eventually falling into a double bind.

### 4.3. Upon Approaching Graduation

By the time participants reached their final undergraduate year, their identity trajectories had clearly diverged. The third Q sort, administered just one month before graduation, showed that the gap between students had widened considerably. The dominant perspective within this cohort reflected a confident dual identity anchored in engineering, while another distinct viewpoint illustrated a deep self-identification crisis.

#### 4.3.1. Factor 1: Engineering-Dominant Integrators

Factor 1 accounted for 38% of the variance with 14 participants loading onto it. Within this perspective, the engineering orientation had now solidified into a primary professional identity. These participants identified strongly as engineering majors and planned to pursue careers exclusively in engineering fields (5, +4; 34, +3; 38, +3). Accordingly, they prioritized engineering in their daily study routines (11, +3).

However, unlike in earlier stages when English was sometimes regarded as less important, these students developed a much stronger appreciation for their English background (29, +2). They endorsed the unique value of the “English + Engineering” combination and felt that it made them more competitive (19, +4), and believed it enhanced their problem-solving abilities (31, +2). Notably, they took great pride in holding a dual degree compared to their single-major peers (22, +3). Interview data suggest that participants largely attributed this identity orientation to their recent experiences in the job market and graduate school applications. Rather than viewing English as merely a communication tool, they came to see it as a key advantage. Some students reflected on this shift during the interview:

“When I attended my postgraduate admission interviews, nearly every interviewer showed strong interest in my English and engineering background. They asked how I managed language learning within a demanding technical program and how the two fields connected. I realized that English gave me extra attention and a unique edge. I no longer see it as just a tool for reading manuals. It has become an indispensable part of my professional identity, distinguishing me from graduates who only major in engineering.”

(Q3)

“Honestly, I once disliked English because it felt useless and took time away from engineering. I just wanted to graduate and find an engineering job. But during job hunting, my English background helped me secure a starting salary 10% higher than what pure engineering graduates received. At that moment, I truly embraced my English identity and felt genuine gratitude for all the language training.”

(Q18)

These narratives illustrate that their interdisciplinary identity was reinforced by external validation. The integration of linguistic and technical competencies resolved their prior identity conflicts and fostered a confident interdisciplinary professional self.

#### 4.3.2. Factor 2: Stuck Negotiators in Identity Crisis

Factor 2 accounted for 13% of the variance and was defined by 4 students, representing a small but highly vulnerable identity position. Unlike their peers who had consolidated a clear professional path, these students remained caught in a severe identity crisis as graduation approached. Their defining feature was overwhelming anxiety about their dual-degree experience and future prospects (23, +4; 25, +4).

These students struggled to balance the demands of both fields and found it impossible to devote equal attention to the two majors (24, +3). Consequently, they failed to develop a sense of belonging to either discipline (18, −4) and remained confused about whether they were language students or engineering students (7, +3). This prolonged tension left them disoriented about their career paths (40, +3). One participant reflected on this fractured identity:

“As graduation approaches, I feel stuck. I have been running between two different academic worlds for years without finding a true anchor. I lack the technical depth to compete with pure engineering students, and my language skills are not strong enough to rely on either. Seeing my classmates succeed with clear identities only makes me more anxious, because I feel like an outsider in both departments with nowhere to go.”

(Q11)

This reflection captures the developmental stagnation of this group. Their persistent inability to integrate the two disciplines coincided with a fragmented perspective of identity at the end of their university journey, which they described as being accompanied by a deep sense of academic defeat.

## 5. Discussion

### 5.1. Discussion of Main Findings

This longitudinal study employed Q methodology to trace the identity profiles of 19 students in an “English + Engineering” dual-degree program over their four-year undergraduate study. Consistent with constructivist and poststructuralist perspectives, the identities of these learners are multifaceted, dynamic, and deeply intertwined with surrounding social contexts (Stets & Burke, 2000; McKinney & Norton, 2024). Specifically, during their first year of undergraduate study, the dominant perspective among participants reflected high expectations for interdisciplinary learning and actively embraced their dual identity, perceiving English and engineering as a mutually reinforcing integrated whole. Meanwhile, an alternative viewpoint displayed an early predisposition to prioritize engineering, regarding English merely as a supplementary tool. At this stage, the students positioned themselves mostly based on personal academic interests and the novelty of the interdisciplinary dual-degree model.

After two years of study, surging academic pressure and practical employment considerations emerged as key factors behind participants’ identity shifts. The primary identity perspective shifted to anchor its core in the engineering major. This transition echoes the findings of Pineda (2024) that students naturally gravitate toward the major with higher expected financial returns when facing severe practical pressures. At the same time, the lack of integration in the curriculum between the two disciplines became a prominent issue that these students linked to their feelings of severe confusion and anxiety. As pointed out by Russell et al. (2008) and Baldry et al. (2018), a superficial combination lacking deep integration makes it difficult for students to build a stable sense of professional belonging. This ultimately leaves them highly vulnerable to losing their balance and developing a sense of defeat while being torn between two different disciplines.

Approaching graduation, compared with Time 1 and Time 2, the engineering-dominant students re-recognized the value of English through positive external feedback during the job search and graduate study applications. They successfully integrated their language advantages into their engineering technology background to form a confident and competitive interdisciplinary professional identity. However, a distinct, marginalized perspective emerged, highlighting a severe identity crisis among those who struggled to reconcile their dual disciplinary identities. Feeling marginalized within both subject fields, they endured overwhelming pressure in job searches alongside persistent psychological frustration. This stagnation in identity development echoes the caution raised by Apple et al. (2020), who argued that dual-degree training inherently poses distinctive identity-related challenges for learners. Without effective interdisciplinary integration, students tend to feel powerless and adrift in their identity.

The longitudinal design of the present study also reveals some nuanced findings that expand upon previous observations. While Pineda (2024) documented that dual-degree students tend to prioritize the major associated with higher financial returns, our study extends this finding by capturing participants’ identification with the less financially rewarding discipline in their final year. Specifically, during the final phase of identity formation, the prevailing perspective among participants ultimately recognized English as a vital component of their identity profiles. Participants associated this shift with positive external feedback. Such patterns complement the work of Holley (2015) and Wimshurst and Manning (2017), who framed classroom activities and peer interaction as primary avenues for resolving identity conflicts. Our findings further indicate that real-world validation, such as others’ positive interest and recognition, can also act as a powerful facilitator enabling students to reconcile their dual disciplinary identities. Furthermore, this research sheds light on the specific experiences of students in programs combining highly distinct academic fields. English and engineering rely on separate epistemological foundations and pedagogical approaches. Investigating this particular disciplinary combination extends existing scholarship on interdisciplinary programs.

It is also worth noting that this study did not find an identity profile anchored in the English major. Although the program was originally designed to treat the two disciplines equally, English was still marginalized during the combination with engineering. This observation resonates with the concerns articulated by Apple et al. (2020). In the face of the overwhelming dominance of STEM fields in the current job market, foreign languages tend to occupy a disadvantaged position. This invisible hierarchical divide makes it difficult for humanities disciplines to maintain an equal footing in such interdisciplinary combinations.

### 5.2. Practical Implications

Based on the above findings and discussions, we propose the following suggestions to optimize dual degree programs. First, the severe curriculum fragmentation reported by students represents a key area for improvement. The foreign language and engineering schools could collaborate to develop genuinely integrated interdisciplinary courses, such as the scientific and technological English literature and international engineering communication practice. Importantly, these courses should position English proficiency as a core component of interdisciplinary competence rather than a subordinate tool for engineering. Clear credit weighting and independent learning objectives for the English component can help prevent the discipline from being marginalized within the dual-degree framework. Second, targeted support may help alleviate the pervasive anxiety over market recognition. The program committee may roll out specialized career guidance at an early stage, and deepen university-enterprise partnerships to demonstrate the distinct competitive advantages of the English-engineering combination to students. Concrete exposure to real industry scenarios can help students recognize the practical value of both disciplines, thereby reinforcing their interdisciplinary recognition. Finally, universities may establish a dynamic tracking mechanism covering students’ academic progress and emotional well-being. For students who exhibit noticeable imbalance in academic effort allocation or signs of identity self-doubt, academic mentors and psychological counselors should deliver timely and targeted interventions to prevent identity crisis.

### 5.3. Limitations and Future Research

This research also has some limitations. First, the study only tracked 19 students from a single university in China. Therefore, the findings might not be fully generalizable to students in other types of institutions or those enrolled in different interdisciplinary dual degree programs. Future research could recruit participants from diverse educational backgrounds. Second, although the study covered the entire four years of undergraduate education, identity construction remains an ongoing journey. The current research does not capture how the identities of these students might continue to shift after they formally enter the workplace or pursue postgraduate studies. Future studies could extend the tracking timeframe into their early career stages to provide a more comprehensive picture of interdisciplinary identity evolution. Third, as noted in the methodology, out of the 22 students who initially participated, three students withdrew from the longitudinal tracking midway, and their incomplete data, therefore, were excluded from analyses of overall identity profile trajectories. Nevertheless, the experiences of these students remain significant, as they may have confronted particular disciplinary conflicts and academic pressures within the dual-degree learning. Future research would benefit from capturing the voices of those who drop out, such as through case studies, thereby offering a more holistic view of interdisciplinary learning experiences.

## 6. Conclusions

This study employed Q methodology to conduct a longitudinal tracking of identity development among 19 “English + Engineering” dual-degree learners, with data collected at three time points from the first academic year to the final year. It reveals that the dominant identity trajectory within this cohort evolved from pluralistic exploration to a singular focus on the engineering major throughout their undergraduate journey, with the English professional identity being gradually marginalized. Meanwhile, a severe identity crisis that emerged in the final year highlights a vulnerable identity position that cannot be ignored. These evolving trajectories reflect how participants navigated and made sense of internal and external factors such as curriculum design, job market orientation, and personal interests. These findings offer actionable insights for developing integrated interdisciplinary dual-degree curricula and providing targeted academic and psychological support for students in comparable mixed-discipline programs.

## Figures and Tables

**Table 1:** Factor extraction.

	Time 1		Time 2		Time 3	
	Factor 1	Factor 2	Factor 1	Factor 2	Factor 1	Factor 2
Eigenvalue	7.15	3.54	9.50	3.16	7.42	2.33
Numbers of significant loaders	10	7	11	7	14	4
% explained variance	29%	28%	38%	29%	38%	13%

## Data Availability

Data available on request due to restrictions.

## Appendix A

**Table A1:** Q-Sort Values for Each Statement in Factor Arrays at Three Timepoints.

No.	Q Statement	Time 1		Time 2		Time 3	
		Factor1	Factor2	Factor1	Factor2	Factor1	Factor2
1	I recognize the unique value of integrating English and Engineering as an interdisciplinary program.	4	2	2	3	1	1
2	I voluntarily chose the “English + Engineering” dual degree learning model based on my personal interests and development needs.	2	0	1	1	1	2
3	Dual degree learning can meet my diverse development needs.	4	2	0	0	1	0
4	I am more inclined to identify myself as an English major.	−4	−4	−3	0	−2	1
5	I am more inclined to identify myself as an engineering major.	−3	4	4	1	4	−2
6	I consider myself both an English major and an engineering major.	3	−3	−3	1	0	−3
7	I am unsure which major I truly belong in.	−3	−2	−2	−2	−4	3
8	I perform better in English than in Engineering, whether in academic coursework or practical application	0	1	3	2	0	2
9	I perform better in Engineering than in English, whether in academic coursework or practical application	−3	−1	−1	0	−3	0
10	I spend more after-class time on English than on engineering.	−2	−3	−4	−4	−3	−1
11	I spend more after-class time on engineering than on English.	2	4	4	0	3	2
12	I follow news and trends related to English more closely than those related to Engineering, such as graduate school opportunities and career prospects.	0	−1	−2	0	−1	0
13	I follow news and trends related to Engineering more closely than those related to English, such as graduate school opportunities and career prospects.	−1	3	2	−1	2	−3
14	I participated in more English-focused extracurricular activities than engineering ones.	0	0	1	−1	2	1
15	I participated in more Engineering-focused extracurricular activities than English ones.	−3	1	0	−1	0	−4
16	I devote more attention to my Engineering coursework than to my English coursework.	2	3	2	0	2	1
17	I devote more attention to my English coursework than to my Engineering coursework.	−1	1	0	−3	−3	−2
18	I have a sense of belonging to both of my current majors.	1	−2	−2	−3	1	−4
19	I believe that dual degree learning makes me more competitive than students with a single major.	3	−1	0	−2	4	1
20	The dual degree program has helped me build networks across different fields.	1	−1	−1	−1	0	2
21	My English proficiency and engineering competencies have achieved synchronous development.	0	−3	−4	−2	−1	−1
22	I feel proud to have a dual-major learning experience.	3	0	−1	0	3	0
23	I feel anxious or stressed when facing the learning pressure of the two majors or switching between the two identity positions.	−1	−1	−1	3	−1	4
24	I find it difficult to give equal attention and commitment to both majors	1	3	2	2	0	3
25	I worry about whether a dual degree will be valued in the job market.	0	1	3	3	−2	4
26	I have never considered switching programs or dropping either of my current majors.	−4	−1	−2	0	−2	1
27	I can adapt my study approach and way of thinking to suit the demands of each major.	1	0	0	−1	−1	0
28	I can draw flexibly on knowledge from both majors depending on what a given situation requires.	0	1	−1	−4	0	−3
29	I believe English can support my Engineering studies.	2	0	1	4	0	0
30	I believe Engineering provides concrete, real-world contexts for applying English, which in turn enhances my English learning.	1	1	0	1	1	−1
31	I believe that interdisciplinary learning helps cultivate my comprehensive learning ability and the capacity to tackle complex problems.	3	2	1	2	2	3
32	Studying across both the humanities and sciences has shaped a more multifaceted personality and a more versatile way of thinking.	0	0	0	1	0	−3
33	I actively seek out interdisciplinary opportunities where I can integrate and apply both my English and Engineering knowledge.	−2	0	−1	3	−1	0
34	I plan to pursue postgraduate studies in a field related to the engineering major.	1	3	3	2	3	−1
35	I plan to pursue postgraduate studies in a field related to the English major.	−2	−4	−3	−3	−4	−2
36	I plan to pursue graduate studies in an interdisciplinary field that draws on the strengths of both degrees.	−2	−2	0	−1	−2	0
37	I hope to work in a language-related field.	−1	−3	−3	−3	−3	−1
38	I hope to work in an engineering or technology-related field.	−1	2	3	−2	3	−2
39	I hope to apply knowledge from both majors in my future career and work across disciplines.	0	0	1	1	1	−1
40	I currently feel uncertain about my future direction and have not yet developed a clear career plan.	−1	−2	1	4	−1	3

## Appendix B

### Interview Prompts

Hi, here is a copy of your completed Q sort.

Review of the sorting experience
  - How do you feel about the sorting experience in general?
  - Before I move on to my questions, is there anything you’d like to ask me?
Retrospect to the Q-sort result
  - You put X at the most agree position. Could you elaborate on why? Anything that might have led you to think this way?
  - You ranked Statement Y as the one you most disagree with. Could you share your thoughts on this?
  - You did not express strong opinions on Statement Z and placed it in the middle. Could you share your thoughts on this statement?
Conclusion
  - Do you have any additional thoughts, feelings, or suggestions to share?

Thank you. If you come up with anything later, please feel free to contact me.

## Appendix C

**Table A2:** The Shifting Identity Profiles of the 19 Participants.

	Time 1	Time 2	Time 3
Q1	Factor 1	Factor 1	Factor 2
Q2	Factor 1	Factor 2	Factor 1
Q3	Factor 2	Factor 1	Factor 1
Q4	Factor 1	Factor 1	Factor 2
Q5	Factor 2	Factor 1	Factor 1
Q6	Factor 1	Factor 2	Factor 1
Q7	Factor 1	Factor 2	Factor 1
Q8	Factor 2	Factor 2	Factor 1
Q9	Factor 1	Factor 1	Non-significant
Q10	Factor 2	Factor 1	Factor 1
Q11	Factor 1	Factor 2	Factor 2
Q12	Factor 2	Factor 1	Factor 1
Q13	Confounding	Factor 2	Factor 1
Q14	Factor 1	Factor 1	Factor 1
Q15	Factor 2	Factor 1	Factor 1
Q16	Confounding	Factor 1	Factor 1
Q17	Factor 1	Factor 2	Factor 2
Q18	Factor 1	Non-significant	Factor 1
Q19	Factor 2	Factor 1	Factor 1
